# Partial eNOS deficiency causes spontaneous thrombotic cerebral infarction, amyloid angiopathy and cognitive impairment

**DOI:** 10.1186/s13024-015-0020-0

**Published:** 2015-06-24

**Authors:** Xing-Lin Tan, Yue-Qiang Xue, Tao Ma, Xiaofang Wang, Jing Jing Li, Lubin Lan, Kafait U. Malik, Michael P. McDonald, Alejandro M. Dopico, Francesca-Fang Liao

**Affiliations:** Departments of Pharmacology, University of Tennessee Health Science Center, 874 Union Avenue/Crowe 401, Memphis, TN 38163 USA; Neurology & Neurobiology, University of Tennessee Health Science Center, 874 Union Avenue/Crowe 401, Memphis, TN 38163 USA; Anatomy & Neurobiology, University of Tennessee Health Science Center, 874 Union Avenue/Crowe 401, Memphis, TN 38163 USA; Department of Neurology, Wuxi Second People Hospital of Nanjing Medical University, 68 Zhongshan Road, Wuxi, Jiangsu province 214002 PR China; Department of Cardiology, The First Affiliated Hospital, Zhengzhou University, No.1 Jianshe road, Zhengzhou, Henan province 450052 PR China

**Keywords:** Endothelial nitric oxide synthase (eNOS), Cerebral chronic hypoperfusion, Cerebral dysfunction, Thrombosis, Microvessel occlusion, Cerebral microinfarction, Vascular dementia, Cerebral amyloid angiopathy (CAA)

## Abstract

**Background:**

Cerebral infarction due to thrombosis leads to the most common type of stroke and a likely cause of age-related cognitive decline and dementia. Endothelial nitric oxide synthase (eNOS) generates NO, which plays a crucial role in maintaining vascular function and exerting an antithrombotic action. Reduced eNOS expression and eNOS polymorphisms have been associated with stroke and Alzheimer’s disease (AD), the most common type of dementia associated with neurovascular dysfunction. However, direct proof of such association is lacking. Since there are no reports of complete eNOS deficiency in humans, we used heterozygous eNOS^+/-^ mice to mimic partial deficiency of eNOS, and determine its impact on cerebrovascular pathology and perfusion of cerebral vessels.

**Results:**

Combining cerebral angiography with immunohistochemistry, we found thrombotic cerebral infarctions in eNOS^+/-^ mice as early as 3–6 months of age but not in eNOS^+/+^ mice at any age. Remarkably, vascular occlusions in eNOS^+/-^ mice were found almost exclusively in three areas: temporoparietal and retrosplenial granular cortexes, and hippocampus this distribution precisely matching the hypoperfused areas identified in preclinical AD patients. Moreover, progressive cerebral amyloid angiopaphy (CAA), blood brain barrier (BBB) breakdown, and cognitive impairment were also detected in aged eNOS^+/-^ mice.

**Conclusions:**

These data provide for the first time the evidence that partial eNOS deficiency results in spontaneous thrombotic cerebral infarctions that increase with age, leading to progressive CAA and cognitive impairments. We thus conclude that eNOS^+/-^ mouse may represent an ideal model of ischemic stroke to address early and progressive damage in spontaneously-evolving chronic cerebral ischemia and thus, study vascular mechanisms contributing to vascular dementia and AD.

**Electronic supplementary material:**

The online version of this article (doi:10.1186/s13024-015-0020-0) contains supplementary material, which is available to authorized users.

## Background

Nitric oxide (NO) synthesized in endothelial cells from L-arginine by endothelial nitric oxide synthase (eNOS) is the major source of NO in brain vessels and an important signaling molecule in vasculogenesis [1–3], cerebral blood flow regulation [3, 4], atherosclerosis and thrombosis [5–8], and amyloid-beta (Aβ) production [9–12]. Thus, intact endothelial function via NO confers key homeostatic balance in normal brain physiology, which raises the possibility that endothelial dysfunction associated with deficiency of eNOS and endothelial NO causes cerebrovascular pathology and neurological disease. Indeed, eNOS gene polymorphisms have been reported to be associated with carotid atherosclerosis [13], ischemic heart and brain [14–16] and possibly, with Alzheimer’s disease (AD) [17]. Correlations between eNOS polymorphisms and decreased NO bioavailability or endothelial dysfunction have also been reported [13, 18, 19], and reduced eNOS expression has been recently found in AD brain [20].

The importance of eNOS in the cardiovascular system was demonstrated in animal models [21]. Targeted deletion of eNOS was shown to cause spontaneous hypertension and exacerbate stroke outcome [3, 4, 21, 22]. In addition, multiple hematologic abnormalities [5, 7], cerebral arteriolar hypertrophy [4], renal thrombotic microangiopathy [8] and myocardial infarction [2, 6] have all been reported in eNOS-deficient mice. Remarkably, neither spontaneously evolving microinfactions nor gross anatomical/histological changes in brain vasculature were observed in these mice [4, 22]. eNOS polymorphisms and haplotypes, however, have been reportedly associated with increased risk of cerebral small-vessel disease as well as silent brain infarction in humans [14, 15]. It should be underscored that the vast majority of work revealing pivotal roles of eNOS in pathophysiology has been conducted on young eNOS^-/-^ mice.

Remarkably, there are no reports of complete eNOS deficiency in humans, caution should be taken when extrapolating conclusions to humans based on data obtained in eNOS^-/-^ mice. Heterozygous eNOS^+/-^ mice, however, constitute an ideal model to begin to explore a possible role of partial eNOS deficiency in neurovascular pathology. Moreover, since aging is an important risk factor for cerebrovascular dysfunction and AD [23–26], it remains critical to determine whether aged eNOS^+/-^ mice present histological and/or functional deficits that may be conclusive to stroke and AD. To address these questions, we combined immunohistochemistry with cerebral fluorescein isothiocyanate (FITC)-dextran microangiography and thus identify potential changes in both brain tissue and vessels in aged (18 months) and young (6 months) eNOS^+/-^ when compared with littermate eNOS^+/+^ mice.

## Results

### eNOS expression, blood pressure, and cerebral microvessel density

**Fig. 1:**
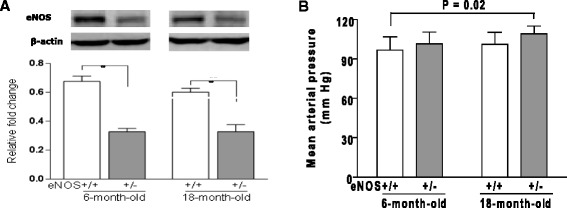
**a** Reduced eNOS protein expression in heterozygous mice. eNOS immunoblotting of 20 μg of total brain lysates. **P < 0.01. *n* = 4 animals of each genotype. Bars represent mean ± s.e.m. **b** Mean arterial pressure (MAP) were calculated based on the measured systolic (SBP), diastolic (DBP). eNOS^+/-^ mice have a propensity to develop hypertension but blood pressure is within normal range compared to age-matched wild-type eNOS^+/+^ mice. *P* = 0.02 by Student’s *t-*test. *n* = 10 animals of each genotype. Bars represent mean ± s.e.m

Consistent with previous reports [4, 22], both young (6 months) and aged (18 months) eNOS^+/-^ mice had lower levels of eNOS protein in brain tissue and a normal range of blood pressure (Fig. 1a and b, respectively). Interestingly, immunohistochemistry showed a higher microvessel density in hippocampus but not in cerebral neocortex in aged eNOS^+/-^ mice when compared with their littermate wild-type (eNOS^+/+^) or young eNOS^+/-^ mice (Additional file 1: Figure S1).

### Spontaneous cerebral thrombosis and microinfarctions

**Fig. 2:**
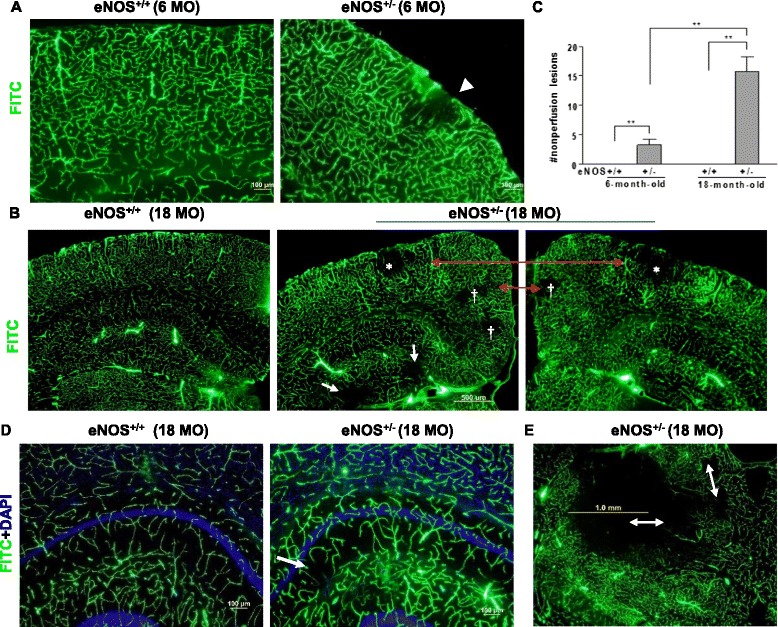
Multiple cerebral occluded lesions in eNOS^+/-^ mice. **a**, **b** Representative FITC-dextran angiographic micrographs in young (**a**) and aged eNOS mice (**b**). Arrows indicate infarctions in hippocampus; red double arrows, bilateral lesions; asterisks, parietal association cortex; daggers, retrosplenial granular cortex; arrowheads, temporal association/temporoparietal cortexes. Scale bars: 100 μm (**a**) and 500 μm (**b**). **c** Quantification of the number of cerebral nonperfusion lesions/infarctions per mouse. **P < 0.0005. *n* = 4–6 mice each genotype. Bars represent mean ± s.e.m. **d** Representative FITC-dextran angiographic micrographs with DAPI counterstaining. Scale bars: 100 μm. White arrow indicates a nonperfusion lesion. **e** Representative angiograph shows multiple cerebral occluded lesions in thalamus (double arrows). Scale bar: 1 mm

To evaluate the functionality of the hyperplasic vessels found in the aged eNOS^+/-^ mice, cerebral fluorescent angiography was performed, which confirmed the immunohistochemical findings of increased microvascular density in hippocampus. Remarkably, angiography revealed multiple cortical/subcortical nonperfusion areas in both young and aged eNOS^+/-^ mice which were not found in littermate eNOS^+/+^ (Fig. 2a, b). Moreover, the nonperfusion areas were more frequent in aged than in young eNOS^+/-^ mice (Fig. 2c). It must be underscored that we did not observe nonperfusion areas in eNOS^+/+^ mice up to 24 months of age. Strikingly, most nonperfusion lesions in aged eNOS^+/-^ mice were bilateral (Fig. 2b). These lesions were not uniformly located throughout the brain but primarily restricted to rather defined areas (in frequency order): parietal association, temporal association and retrosplenial granular cortexes, hippocampus and thalamus.

In addition, we observed markedly higher FITC signal in the microvessels surrounding the larger nonperfusion areas (Fig. 2d), suggesting a compensatory vascular dilation presumably due to more severe ischemia in these older animals than in younger mice. While most nonperfusion lesions were small (diameter of 100–500 μm) and the cortical lesions rarely extended beyond the outer half of cortex, some thalamic lesions were up to 1.5 mm in diameter (Fig. 2e). Interestingly, these nonperfusion lesions were rarely found in sections without hippocampal involvement (i.e., the anterior portion of the brain), underscoring the particular vulnerability of hippocampal formation and function to partial eNOS deficiency.

**Fig. 3:**
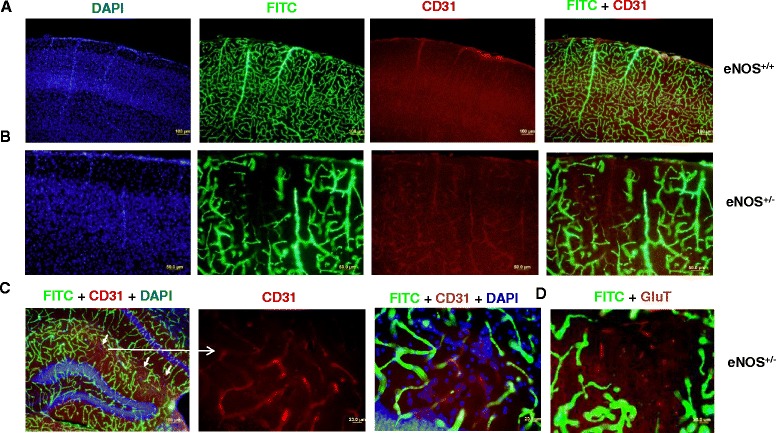
Immunohistochemistry of CD31/GluT1 and DAPI on FITC-dextran-perfused brain sections in 18-month old eNOS^+/+^ (**a**) and eNOS^+/-^ (**b**) mice. **a** Parietal cortex; Scale bar: 100 μm. **b** Parietal cortex; Scale bar: 50 μm. **c** Hippocampus; Scale bars: 100 μm (left panel) and 20 μm (right panels). **d** Representative images of GLuT1 and DAPI on FITC-dextran-perfused brain sections corresponding to a nonperfusion lesion. Scale bar: 20 μm

To determine whether nonperfusion areas were due to vessel obstruction or an actual loss of blood vessels, we combined cerebral fluorescent angiography with immunofluorescent staining using CD31 and GluT1. Owing to thick brain sections (100 um) from FITC-dextran perfused brains, immunostaining signals were much weaker (Fig. 3a and b) as compared to formalin-fixed tissue sections (40 μm). To our surprise, CD31**-**positive vessels with diameters of 5–20 μm were largely present within nonperfusion areas (Fig. 3b, c), suggesting that vessel loss does not critically contribute to the nonperfused areas found in eNOS^+/-^ mice. This was confirmed by another vascular marker GluT1 (Fig. 3d), though displaying a discontinued profile. These findings are consistent with the fact that microvessel density remains unchanged in neocortex in aged eNOS^+/-^ mice.

**Fig. 4:**
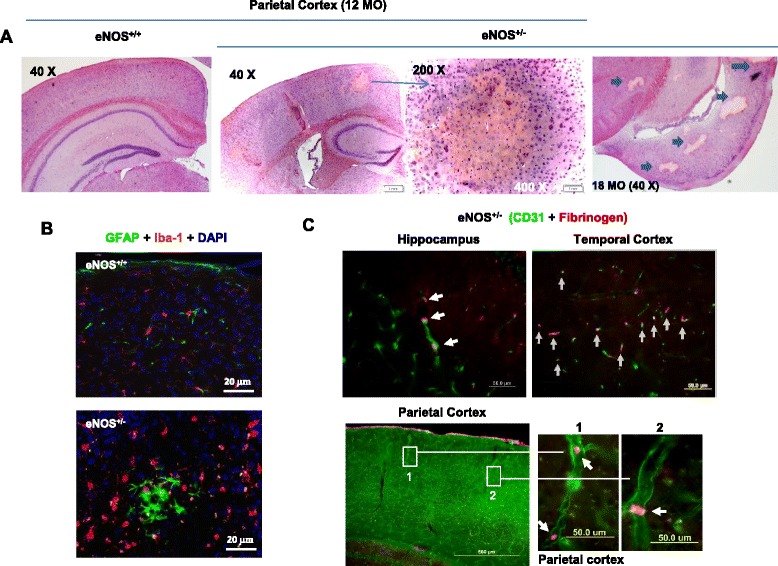
Molecular and cellular characteristics of the cerebral occluded lesions in parietal cortex in 18 months eNOS^+/-^ mice: partial eNOS deficiency causes cerebral thrombosis. **a** Representative images of H & E Staining (6 μm coronal sections). **b** GFAP/Iba-1 immunohistochemistry on 12 months eNOS brains. Scale bars: 20 μm. **c** Representative images of fibrinogen/fibrin (red) and/or CD31 (green) immunohistochemistry in 18-month-old eNOS^+/-^ mice. White arrows indicate a string of fibrinogen/fibrin-positive signals lining with CD31-positive vessels (upper) and individual fibrinogen clots inside the arterioles (lower, insets). Scale bars: Upper panels: 50 μm; Lower panel: 500 μm (insets, 50 μm)

In coronal sections, H & E staining revealed markedly reduced cellularity and necrosis in the core of lesion in eNOS^+/-^ mice (12 months) (Fig. 4a), despite marked gliosis (GFAP/astroglias and Iba-1/microglias) (Fig. 4b). The nonperfusion areas detected in eNOS^+/-^ brains followed all criteria required by the accepted definition of microinfarcts: sharply delineated microscopic regions of cellular death or tissue necrosis, sometimes with cavitation [27]. Moreover, different stages of microinfarcts based on the H & E histology were detected in eNOS^+/-^ brain at various ages; the multiple pale lesions detected in older mice most likely revealed chronic lesions of late staged infarcts, presumably with the damaged tissue phagocytized (Fig. 4a, right panel). No similar lesions at any stage were detected in eNOS^+/+^ mice at all ages.

Next, to elucidate the cause(s) of vascular obstruction, we determined the presence of intravascular thrombus. Immunohistochemistry of fibrinogen/fibrin confirmed multiple cortical and subcortical intravascular microthrombi blocking the vessels in aged eNOS^+/-^ mice (Fig. 4c) and, to a lesser degree, in young eNOS^+/-^ mice. In sharp contrast, microthrombi were never detected in either aged or young eNOS^+/+^ mice (data not shown). Some intravascular thrombi were much smaller than the lumen of the arterioles, but they were not washed out by transcardial perfusion during tissue preparation (Fig. 4c, right lower panels), indicating that these in situ mural thrombi were due to thrombosis rather than thromboembolism. The presence of continuous strings of fibrinogen-positive signal along the vessels also supports the occurrence of thrombosis.

### CAA, blood brain barrier (BBB) breakdown, and cognitive dysfunction

**Fig. 5:**
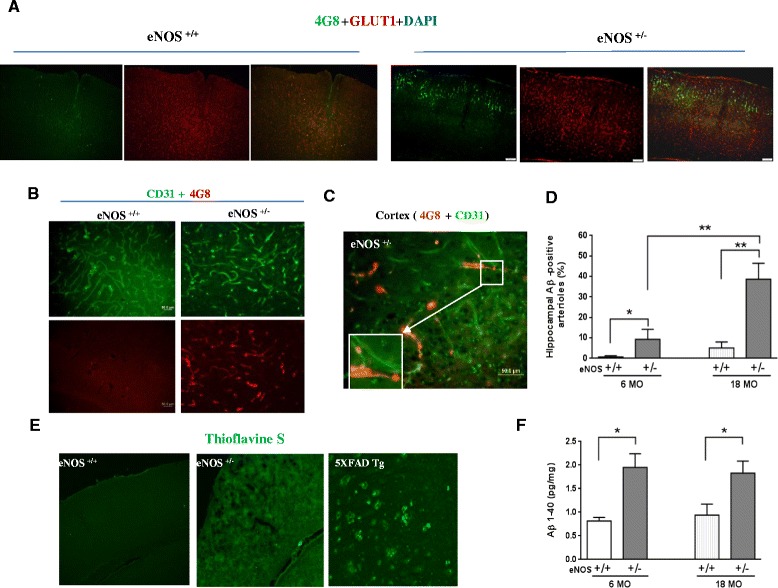
Partial eNOS deficiency promotes CAA. **a** Representative images of anti-Aβ (4G8, green) and GLUT1 (green) immunohistochemistry on cerebral cortex (100 X). **b**, **c** Representative images of anti-Aβ (4G8, red) and CD31 (green) immunohistochemistry on cerebral cortex (Scale bars: 100 μm) (B) and on hippocampal fissure (C, Scale bars: 50 μm). **d** Quantification of Aβ-positive arterioles in hippocampal fissure. *P = 0.01; **P < 0.001. *n* = 5–6 mice each genotype. **e** Representative images of double immunohistochemistry in hippocampal fissure. Scale bars, 50 μm. E) Representative images of Thioflavine S images taken from cortex. **f** Quantification of forebrain soluble Aβ40 levels by ELISA (Life Technologies, CA, Cat^#^KMB3481). *n* = 5 each genotype

We and others have found BACE1 upregulation and elevated levels of soluble Aβ in young adult eNOS^-/-^ brain (4 months) [9–12]. We then sought to determine amyloid deposition in older mice. In aged eNOS^+/-^ mice, we found significant cerebral amyloid angiopathy (CAA) that showed dense Aβ deposits within and surrounding the vessel walls of the arterioles in cerebral pia mater and parenchyma (Fig. 5a-c) as well as in the hippocampi (Fig. 5d). CAA was also found in the capillaries reaching the temporoparietal cortex, hippocampal formation, amygdala, hypothalamus, and striatum (data not shown). In contrast, CAA was only occasionally found in aged eNOS^+/+^. Moreover, while young eNOS^+/-^ mice (8 months) displayed mild CAA, no evidence of CAA could be found in young eNOS^+/+^ mice (data not shown). Detection of Aβ aggregation by Thioflavine S staining only revealed a diffusive signal in the brains of eNOS^+/-^ mice, rather than as focal plaques as in 5XFAD mice (Fig. 5e). Quantification of Aβ immunoreactivity by ELISA in forebrain indicated a two-fold increase in soluble Aβ40 starting from young aged eNOS^+/-^ mice (4–6 months) when compared to littermate controls (Fig. 5f).

**Fig. 6:**
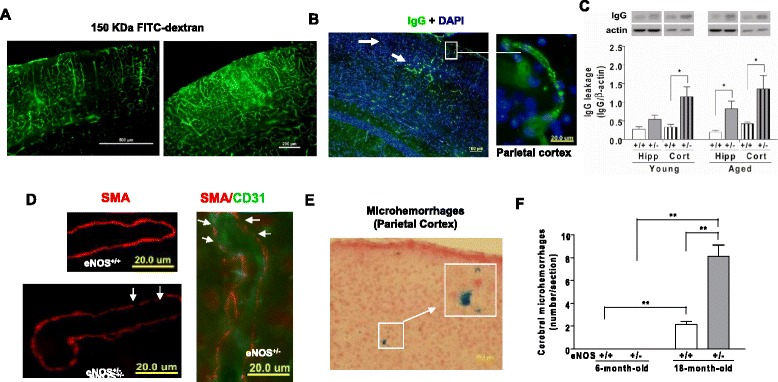
Partial eNOS deficiency causes cerebral BBB breakdown and smooth muscle degeneration. **a** Angiography 60 min after injection of FITC-dextran (150 Kd), showing diffusive fluorescent signal as an indication of leakage vessels surrounding the occluded area. **b** Representative images with DAPI counterstaining shows parietotemporal cortical vascular extravasations of serum IgG (green, white arrowheads) in 18-month-old eNOS^+/-^ mice. Scale bars: left panel 100 μm (inset, 20 μm). **c** Quantification of extravasated mouse IgG based on anti-mouse IgG immunoblot analysis. Note less expression of beta actin in hippocampus (Hipp) than in cortex (Cort). Values are normalized to beta actin (β-actin out of 20 μg total proteins) and expressed as mean ± s.e.m. *P < 0.05. *n* = 4 animals each genotype. **d** Immunofluorescent staining with anti-SMA (red) and/or anti-CD31 (green) antibodies in aged (18 months) wild-type and heterozygote eNOS mice. Left panels: arterioles in hippocampal fissure; right panel: parietal cortex. Arrows indicate degeneration of smooth muscle cells. Scale bars, 20 μm. **e** Representative images of Prussian blue staining (blue) in cortex in aged eNOS^+/-^ mice. Scale bars, 20 μm. **f** Quantification of microhemorrhages based on Prussian blue staining **P < 0.0001. *n* = 5 mice each genotype. Bars represent mean ± s.e.m

To better understand the consequence of cerebral thrombosis and CAA, we next studied the leakage of endogenous blood-derived protein in the brain. As shown in the cortex, aged eNOS^+/-^ mice had localized extravasations of FITC-dextran (150 Kd, Fig. 6a, right panel) and serum IgG (Fig. 6b, c, Additional file 1: Figure S2). Locations and distribution of serum IgG extravasation were well matched with those of nonperfusion areas as demonstrated by angiography in aged eNOS^+/-^ mice, consistent with the notion that thrombotic cerebral infarctions and localized BBB breakdown are pathophysiologically related in the eNOS^+/-^ mouse. Moreover, vascular smooth muscle cell degeneration (Fig. 6d) and a greater number of cerebral microhemorrhages scattered over bilateral hemispheres (Fig. 6e, f).

**Fig. 7:**
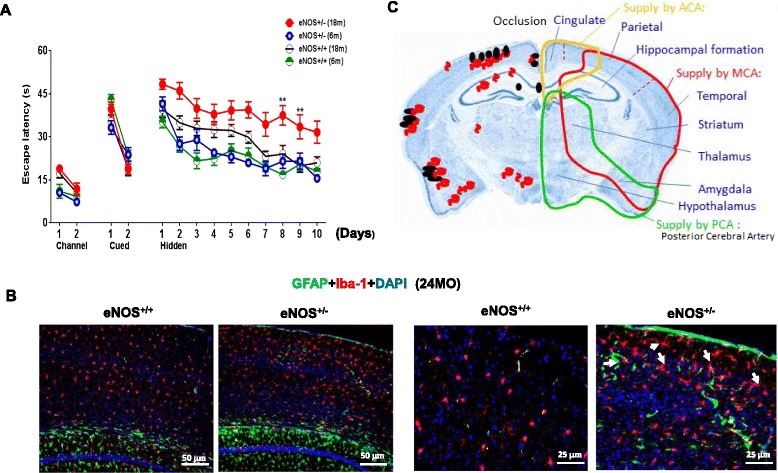
**a** Cognitive functions in heterozygous eNOS knockout (eNOS^+/-^) mice. Spatial learning and memory evaluated by water maze tests reveals no significant differences between young (6-month-old) eNOS^+/-^ mice and their littermate wild-type (eNOS^+/+^) mice (*n* = 10 each genotype), but severe impairment in aged eNOS^+/-^ mice when compared to eNOS^+/+^ mice (*n* = 18 each). Data are presented as means ± SEM. **P <0.005 when eNOS^+/-^ 18months group was compared to that of the littermate eNOS^+/+^ mice. **b** Representative images of GFAP and Iba-1 double immunohistochemistry. White arrowheads indicate activated microglias. Scale bars: 50 μm form the left panels and 25 μm for the right panels. **c** Schematic summary of the relative locations of the identified occluded lesions (black) and CAA (red) in old eNOS^+/-^ mice

Cerebral thrombosis or microinfarctions likely contribute to cognitive decline. Recently, a single cortical microinfarction event induced by penetrating arteriole obstruction was shown to cause cognitive deficits in mice [28]. We therefore hypothesized that eNOS partial deficiency-induced cerebral infarctions have a negative impact on cognitive performance. Consistent with this idea, by water maze test, we found progressive cognitive deficits in old eNOS^+/-^ mice compared to eNOS^+/+^ controls; no significant difference found in young mice (6 months) (Fig. 7a). Moreover, marked neuroinflammation (activated microglias/Iba-1 and astrocytes/GFAP) and neurodegeneration with cortical atrophy (~20 % reduction of cortical thickness) were detected in 24 months) eNOS^+/-^ mice (Fig. 7b).

### Altered molecular profiles

**Fig. 8:**
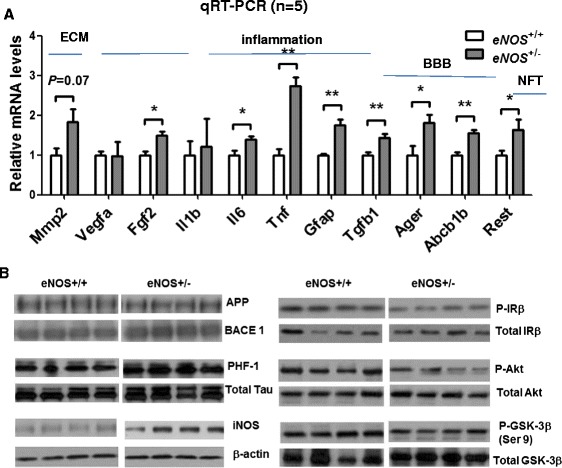
Molecular evidence of neuroinflammation and impaired insulin resistance and BBB transport. **a** Quantitative qRT-PCR of frontal brain tissue. **b** Western blot of frontal brain lysates (20 μg). Gene names used are Mmp2: MMP2; Vegfa: VEGFa; Fgf2: FGF2; Il1b: IL-1 β; Tnf: TNF-1α; Gfap: GFAP; Tgfb1: TGF-1β; Ager: RAGE; Abcb1b: ABCB1B; Rest: REST

To determine molecular changes in eNOS^+/-^ mice, we performed qRT-PCR of frontal brain tissue. When compared with littermate controls, eNOS^+/-^ mice showed progressive changes in genes involved in a variety of neurovascular processes such as angiogenesis, inflammation, and BBB transport. Specifically, we found a significant increase in the mRNAs of bFGF-2 and MMP2 (but not VEGF-A or MMP9 or thrombin), ICAM-1, IL-6, TNF-α and GFAP (but not IL-1β) (Fig. 8a). Interestingly, gene *Ager* (RAGE) is upregulated but not *lrp1* (LRP1). Lipoprotein receptor-related protein (LRP-1) and receptor for advanced glycation end products (RAGE) are the two molecules most closely related to Aβ uptake/clearance and protein transport across the BBB [29–32]. Altered expression of either of these capillary endothelial receptor proteins could indicate a dysfunction of the BBB and its transport regulation of Aβ. The increased expression of Abcb1b (ABCB1b, APC transporter) also strongly suggests a disrupted function in BBB transport. In addition, TGFβ has been extensively studied in terms of its role in Aβ biogenesis and neuroinflammation [33]. It is thus very important to study eNOS-regulated TGFβ, which has thus far been unexplored. Our data show a ~ 2-fold increase in tgfb1 mRNAs in the eNOS^+/-^ mouse brain. This first finding suggests an involvement of TGFβ − mediated pathways in eNOS mice, which warrants future investigation. Finally, biochemical analysis also reveals evidence of increased BACE1 expression and hyperphosphorylated tau as well as CNS insulin resistance in aged eNOS^+/-^ mice (Fig. 8b).

## Discussion

The present study reveals, for the first time, the occurrence of spontaneous cerebral thrombosis at early age in eNOS^+/-^ mice (< 6 months). Moreover, these thrombotic phenomena increase with age, and are accompanied by progressive CAA detectable from 8 months of age and cognitive dysfunction detected at old age (18 months). But, perhaps what is most striking is that we detected multiple occluded areas/lesions with diameters ranging from 0.1 to 1.5 mm in three primary areas (i.e., temporoparietal and retrosplenial granular cortexes as well as hippocampus). Each of these regions matches precisely to the hypoperfused areas identified in pre-clinical AD patients [34–36], which indicates that the partial eNOS-deficient mice is a spontaneous model of chronic hypoperfusion.

Microscopic examination of aging brain sections, particularly those exhibiting cognitive decline, often reveal numerous microinfarctions resulting from small vessel pathologies such as arteriolosclerosis or CAA [27]. Microinfarctions and ischemic stroke can be induced by thrombosis, which are usually associated with increased cellularity due to inflammatory infiltration and gliosis during an acute stage. We observed reduced cellularity in the cores of occluded lesions most of time. Based on the neuropathological definition of sharply delineated microscopic regions of cellular death or tissue necrosis, we believe the occluded lesions are in fact microinfarctions. The pale regions (Fig. 4a, right panel) likely represent chronic lesions (i.e., “old infarcts”), which typically demonstrate cavitation with surrounding fibrillary gliosis. We occasionally detected more freshly looking lesions in the neocortex, which showed an acute ischemic appearance of red neurons and loss of colorful staining quality (not shown). Given the high frequency and widespread distribution pattern in eNOS mice, these microinfarctions predictably contribute to direct disruption of important cognitive networks.

While these results were fascinating, an important question remains unanswered: what causes cerebral thrombosis (and or thrombotic microinfarctions) if there was no detection of significant changes in the blood pressures of eNOS^+/-^ mice at all ages. We favor the hypothesis that chronic partial eNOS deficiency causes endothelial and platelet dysfunction, which leads to thrombotic cerebral infarctions. Indeed, vascular and platelet activity have been found to be compromised in eNOS-deficient mice [37].

Based on our data on the sequential events of thrombosis, CAA and cognitive decline in eNOS^+/-^ mice, we further propose the following theory: that chronic partial eNOS deficiency causes endothelial and platelet dysfunction that ultimately leads to the thrombotic cerebral infarctions and associated BBB breakdown that facilitate Aβ production and impair Aβ clearance [38]. Both of these events promote vascular deposition of Aβ, resulting in vessel tortuosity (Additional file 1: Figure S3) which in turn triggers platelet activation and exacerbates thrombotic cerebral infarction [39]. Although we did not measure the nitric oxide (NO) content in the brain of eNOS^+/-^ mice, reduced levels of systemic NO in all organs were determined based on dose-dependent phenotypes detected in these mice as compared to eNOS^+/+^ and eNOS^-/-^ littermates [40–44], with one exception [45]. Thus, it is generally believed that the phenotypes observed in eNOS-deficient mice are due to the reduced NO levels/functions.

It should be noted that our report is the first to use a mouse model that does not overexpress a mutant amyloid precursor protein (APP) or presenilin (PS1) gene to develop aberrant Aβ generation and deposition within vessel walls (presumably via an eNOS-dependent regulatory mechanism). Moreover, contrary to most FAD models, including a recently established APP-PS1 knock-in model [46], in which amyloid pathology (CAA in particular) causes cerebral hypoperfusion, our model clearly demonstrates that chronic hypoperfusion in fact can induce CAA. The other major differences in our spontaneous eNOS and FAD models are the degree of CAA and the composition of mouse versus human Aβ species. The two-fold increase of Aβ observed in both soluble and diffusive plaques in eNOS mice probably indicate milder CAA when comparing to other FAD models (Fig. 5) [47]. However, we did not observe focal Aβ plaques at 24 months of age in eNOS^+/-^ mice, which is consistent with the notion that endogenous mouse Aβ is less aggregable and do not form plaques. Nevertheless, it may be informative to further compare our eNOS model with those of FAD’s. Since mouse Aβ is less toxic than human’s, it may also be important to cross eNOS-deficient mice with AD mice expressing human FAD APP mutants to study interplay between chronic hypoperfusion and amyloid progression.

**Fig. 9:**
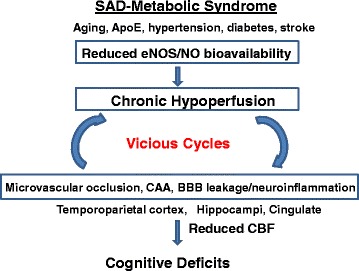
Schematic demonstration of a “vicious cycle” hypothesis. We believe that chronic cerebral hypoperfusion and aberrant generation of amyloid are the two key components forming the vicious cycles for vascular dysfunction and dementia. Compared to ischemia-induced severe hypoperfusion, eNOS-deficiency may result in milder but more chronic hypoperfusion, pointing to a novel pathophysiological mechanism in the vascular deficits of AD that ultimately leads to the aforementioned vicious cycles. SAD: sporadic Alzheimer’s disease; CBF: cerebral blood flow

Cerebral hypoperfusion has been identified as a hallmark in the pre-clinical phase of AD, although whether it plays a causal role in AD pathogenesis remains unclear. Cerebral Hypoperfusion and CAA are both invariant features of dementia and AD [48, 49]. Thus we propose that cerebral hypoperfusion/infarction and CAA form a vicious cycle for vascular dysfunction and, ultimately, dementia (Fig. 9). This hypothesis is supported by the anatomically close vicinity of these two events (Fig. 7a), as well as by the notion that severe CAA is induced by cerebral cortical microinfarctions in humans [50]. Given that FAD mutations were only detected in < 2 % of AD cases, and hypoperfusion was identified in a majority of preclinical AD patients without detectable amyloid pathology, an eNOS^+/-^ model is more representative of sporadic AD cases and is predictably useful for studying early pathogenic mechanisms. Compared to ischemia-induced severe hypoperfusion, eNOS-deficiency may result in milder but more chronic hypoperfusion, suggesting a novel pathophysiological mechanism in the vascular deficits of AD that ultimately leads to the aforementioned vicious cycle.

## Conclusions

Stroke is the third leading cause of death and an important cause of age-related cognitive decline and dementia. However, the development of effective therapies for stroke and vascular dementia has been limited due to the lack of suitable animal models [51]. The only non-induced model that mimics both microvessel and parenchymal changes in human stroke is the spontaneously hypertensive stroke-prone rat. Although some susceptibility genes have been identified [52], this rat line is highly inbred and may be of limited relevance [53]. Consistent with the previous report [22], our data reveal an absence of severe hypertension in eNOS-deficient mice, yet interestingly enough these mice spontaneously develop cerebral infarctions. Therefore, the eNOS^+/-^ mouse may represent a spontaneous cerebral infarction model for stroke and vascular dementia, best fit to understand pathophysiological mechanisms that would assist in developing novel preventative therapeutic strategies. Stroke is an important risk factor for AD [24–26]. Our most striking finding is that partial eNOS deficiency-induced cerebral infarctions match well with the patterns of cerebral blood flow distribution in AD patients [34–36]. This suggests that the eNOS deficient mouse may also represent a model for sporadic AD, which would greatly improve our understanding of AD etiology and ultimately create better prevention and treatment therapies.

## Methods

### Materials and methods

#### Animals

eNOS^+/-^ and littermate eNOS^+/+^ mice were derived from interbreeding eNOS^+/-^ and eNOS^+/+^ mice by using eNOS^-/-^ mice (NOS3^tm1Unc^/J Jackson Laboratory, Bar Harbor ME). Each mouse was genotyped.

### Cerebral fluorescent angiography and tissue collection

Cerebral fluorescent angiography was performed as described in detail elsewhere [54]. Briefly, FITC-dextran (2000 Kda; 0.1 mL 50 mg/mL in double-distilled water; Sigma, St. Louis, MO, USA) was administered intravenously via mouse tail vein. FITC-dextran remained dissolved and free in plasma. Two minutes after injecting FITC-dextran, animals were sacrificed. Brains were rapidly removed and placed in 4 % paraformaldehyde (PFA) in PBS at 4 °C for 24 h and then incubated in 30 % sucrose in PBS for another 24–36 h at 4 °C. Sequential coronal sections (100 μm thick) were cut using a vibratome and kept at −20 °C in an antifreeze solution (ethylene glycol: glycerol: 0.1 M PBS: double-distilled water = 1:1:1:1) until further processing. For the permeability test, we injected FITC-dextran (150 Kda; 0.1 mL 50 mg/mL) via tail vein and perfused mice 60 min after injection before processing for vibratome sections.

### Measurement of blood pressure

Systolic (SBP), diastolic (DBP) and mean arterial pressure (MAP) were measured using a noninvasive tail-cuff method (model XBP 1000, Kent Scientific, Torrington, CT) as described [55]. All measurements were taken in the morning, and the first 5 of 25 measurements taken each day was discarded to obtain data after the mouse was acclimatized to the equipment. Blood pressure was measured for 4 consecutive days, and mean values from individual mice were used for analysis.

### Morris Water Maze Test (MWM)

Spatial learning and memory were examined in a MWM task as we previously described in detail [56]. A video camera was mounted at the height of 180 cm above the center of the maze, and all data were recorded with a computerized video system. Escape latency (finding the submerged escape platform) and path length to find the hidden platform were recorded. On day 9, the probe test was performed by removing the platform and allowing each mouse to swim freely for 60 s. The total length of the swim path during the testing period was recorded. The time that mice spent swimming in the target quadrant (where the platform was located during hidden platform training) was measured. For the probe trials, the number of times the mice crossed where the platform had been located was also measured and calculated.

### Histocytochemistry

Mice were perfused transcardially with ice-cold PBS followed by ice-cold 4 % PFA in PBS. Then, brains were removed from the cranium. After postfixation in 4 % PFA in PBS at 4 °C overnight, brains were incubated in 30 % sucrose in PBS for 24–36 h at 4 °C. Brains were then cut into serial coronal sections (60 μm thick) on a vibratome. The sections were kept at −20 °C in the antifreeze solution until further processing. Perfused or angiographic brain (i.e., without transcardial perfused fixation) sections were treated with 0.3 % triton X-100 for 10 min and blocked with 10 % normal goat serum or 10 % BSA for 60 min at room temperature. Then, samples were incubated with primary antibody diluted in blocking solution overnight at 4 °C. We used the following primary antibodies: beta amyloid 17–24 (4G8) monoclonal antibody (Covance; Cat# SIG-39220; 1:100), rat anti-mouse CD31 (BD BioScience; Cat#553370; 1:50), anti-GluT1 (~54 kDa) rabbit polyclonal antibody (Millipore; Cat#07-1401; 1:100), rabbit anti-mouse fibrinogen/fibrin polyclonal antibody (MyBioSource; MBS315814; 1:700), mouse anti-GFAP (Sigma, G3893; 1:1000), rabbit anti-SMA (ABCAM, ab5694; 1:100), rabbit anti-Iba-1 (WAKO, #019-19741; 1:500), Anti-phospho-IGF-IRβ (Tyr1135/1136)/insulin receptor (IR)-β (Tyr1150/1151) (19H7) and anti-IRβ (Cell Signaling Technology #3024 and #3025), anti-P-Akt (Thr 473) and anti-Akt (Cell Signaling Technology, #9271 and #9272), mouse anti-GSK3 β (Ser9) (Cell Signaling Technology #9336) and anti-GSK-3β (#9315), Tau-5 (Santa Cruz, sc-58860) and goat anti-mouse IgG (Biolegend; Cat# 40531;). Thioflavine S was obtained from Sigma. Sections were washed in PBS and incubated with the following secondary antibodies dilated in blocking solution 60 min at room temperature: Alexa Fluor 594 goat anti-mouse IgG (H + L) (Invitrogen; A11032; 1:500) to detect mouse Aβ, Alexa Fluor 488 or 555 goat anti-rat (Invitrogen; A1106 and A11057, respectively) 1:500 to detect mouse CD31, Alexa Fluor 594 goat anti-rabbit IgG (H + L) (Invitrogen; A11037; 1:500) to detect GluT1 and fibrinogen, and DyLight 488-conjugated AffiniPure donkey anti-goat IgG (H + L) (Jackson ImmunoResearch; Code 705-485-147; 1:500) to detect mouse IgG. Sections were washed in PBS and counterstained with DAPI (Roche Diagnostics, Lot # 70237122, 1:1000). After washing with PBS three times, sections were mounted on microscopy slides and covered with Fluoromount-G (SouthernBiotech, Cat # 0100-01).

### Western blot analysis and quantitative real-time RT-PCR (qRT-PCR)

Western blot analysis was performed as described [10, 12] using 20 μg of total protein lysates freshly prepared from the frontal brain tissue. For qRT-PCR, RNA was prepared using TRIzol (Invitrogen) according to the manufacturer’s instructions. Single-stranded cDNA was synthesized from 1 μg of total RNA using High Capacity cDNA Reverse Transcription kits (Applied Biosystems). Quantitative real-time PCR was performed with RealMasterMix SYBR ROX (5 Prime) according to the manufacturer’s protocols using the same rat and mouse GAPDH primers. Primers for other mouse genes are listed below: Mmp2 taacctggatgccgtcgt/ttcaggtaataagcacccttgaa; Vegfa gttgcctagtgggtggatct/gctacccatccagcctgtt; fgf2 cggctctactgcaagaacg/ tgcttggagttgtagtttgacg; agcttcaggcaggcagtatc/Il1b gtcacagaggatgggctctt; tnfa ccctcacactcagatcatcttct/gctacgacgtgggctacag; gfap tcgagatcgccacctacag/gtctgtacaggaatggtgatgc; TGF1b tggagcaacatgtggaactc/ gtcagcagccggttacca; Ager agcctgggaaggaagcac/ggctgtgatgttctgaccac; abcb1b ccaaattctacatcttggctgac/ttcaaactccatcaccacctc; rest agcaacaaagaaaaggagttgg/acctgggtggccataactg.

### Assessment of cerebral microvessel structure and function

Cerebral blood vessel density was assessed according to the point-counting method [57] on FITC-dextran-perfused sections that were counterstained with DAPI. The number of point intersections of CD31-positive and FITC-dextran-perfused profiles was scored in a 100-point grid, which covered the entire surface of the microscopic field so that surface area was calculated according to the magnification used (×100). Vessel density is expressed as the percentage of brain surface cover with vessels. Measurements were performed in bilateral CA1 regions including oriens layer, pyramidal cell layer and radiatum layer hippocampi in 4 sections taken at 0.6 mm intervals (9–10 sections per brain with 4 sections showing both CA1 region and cerebral neocortex, ranging from Bregma 1.98 to Bregma −3.28 mm). To calculate the number of cerebral infarctions, 9–10 angiographic sections taken at 0.6 mm intervals (ranging from Bregma 1.98 to Bregma −3.28 mm) were assessed using fluorescence microscopy under low magnification (10x), with results being expressed as arbitrary units (total number of infarctions in 9–10 brain sections). CAA and vascular smooth muscle cell injury were determined using CD31 in combination with Aβ and SMA immunofluorescent staining, respectively. The percentage of Aβ positive arterioles in bilateral hippocampal fissures were calculated in 4 hippocampal sections taken at 0.6 mm intervals (9–10 sections per brain ranging from Bregma 1.98 to Bregma −3.28 mm with 4 sections showing hippocampus). Blood–brain barrier breakdown was evaluated by either extravasations of mouse IgG by using immunofluorescent stain or deposition of ferric iron from hemorrhage by using Perl’s Prussian blue staining.

### Statistical analysis

Statistical analyses were performed using unpaired two-tailed or one-tailed Student’s *t*-test. Data are reported as mean ± S.E.M. Statistical significance was set at *P* < 0.05.

## Additional file

Additional file 1: Figure S1. Cerebral microvessel density in eNOS^+/-^ mice. A-C: Representative images of immunofluorescent staining with anti-CD31 (an endothelial cell marker) antibodies showing vascular hyperplasia in hippocampal CA1 region in aged (18-month-old) eNOS^+/-^ mice (A) compared with either their littermate wild-type (eNOS^+/+^) mice (B) or young (6-month-old) eNOS^+/-^ mice (C). Scale bar = 20 μm. D and E) Quantification of CD31-positive microvessel density in 4 sections with both hippocampus and cerebral cortex taken at 0.6 mm intervals in young (6-month-old) and aged mice. Results are expressed as mean ± SEM. **P* < 0.005; ***P* < 0.0005. n = 5–6 animals each genotype. **Figure S2.** Vascular extravasations of serum IgG in 18-month-old eNOS^+/-^ mice. Representative images of mouse IgG immunohistochemistry showing leakage areas framed by white box indicating extravasated mouse serum IgG (green). Scale bars: 200 μm (inset, 50 μm). **Figure S3.** Cerebral infarction and tortuous cortical arterioles in heterozygous eNOS^+/-^ mice. Cerebral fluorescein isothiocyanate (FITC)-dextran (green) angiographic micrograms showing cerebral microinfarcts/nonperfusion areas (A, B, D, and E. asterisks) in parietal and parietotemporal cortexes with or without surrounding vascular dilation and/or hyperplasia (arrows) in aged (18-month-old) and young (6-month-old) eNOS^+/-^ mice. Note that cortical penetrating arterioles are more tortuous in aged eNOS^+/-^ mice (A and B, arrowheads and inset) compared with their littermate wild-type (eNOS^+/+^) mice (C and inset) and young eNOS^+/-^ (E) and eNOS^+/+^ (F and inset) mice. Scale bar = 100 μm.

## Abbreviations

eNOS: Endothelial nitric oxide synthase
CAA: Cerebral amyloid angiography
FAD: Familial Alzheimer’s disease
SAD: Sporadic Alzheimer’s disease
CBF: Cerebral blood flow
GluT1: Glucose Transporter-1
