# Supplementary figures and images for: Partial eNOS deficiency causes spontaneous thrombotic cerebral infarction, amyloid angiopathy and cognitive impairment
Source: Mol Neurodegener. 2015 Jun 24;10:24. doi: 10.1186/s13024-015-0020-0 (PMC4479241; doi:10.1186/s13024-015-0020-0)

Online Figure 1

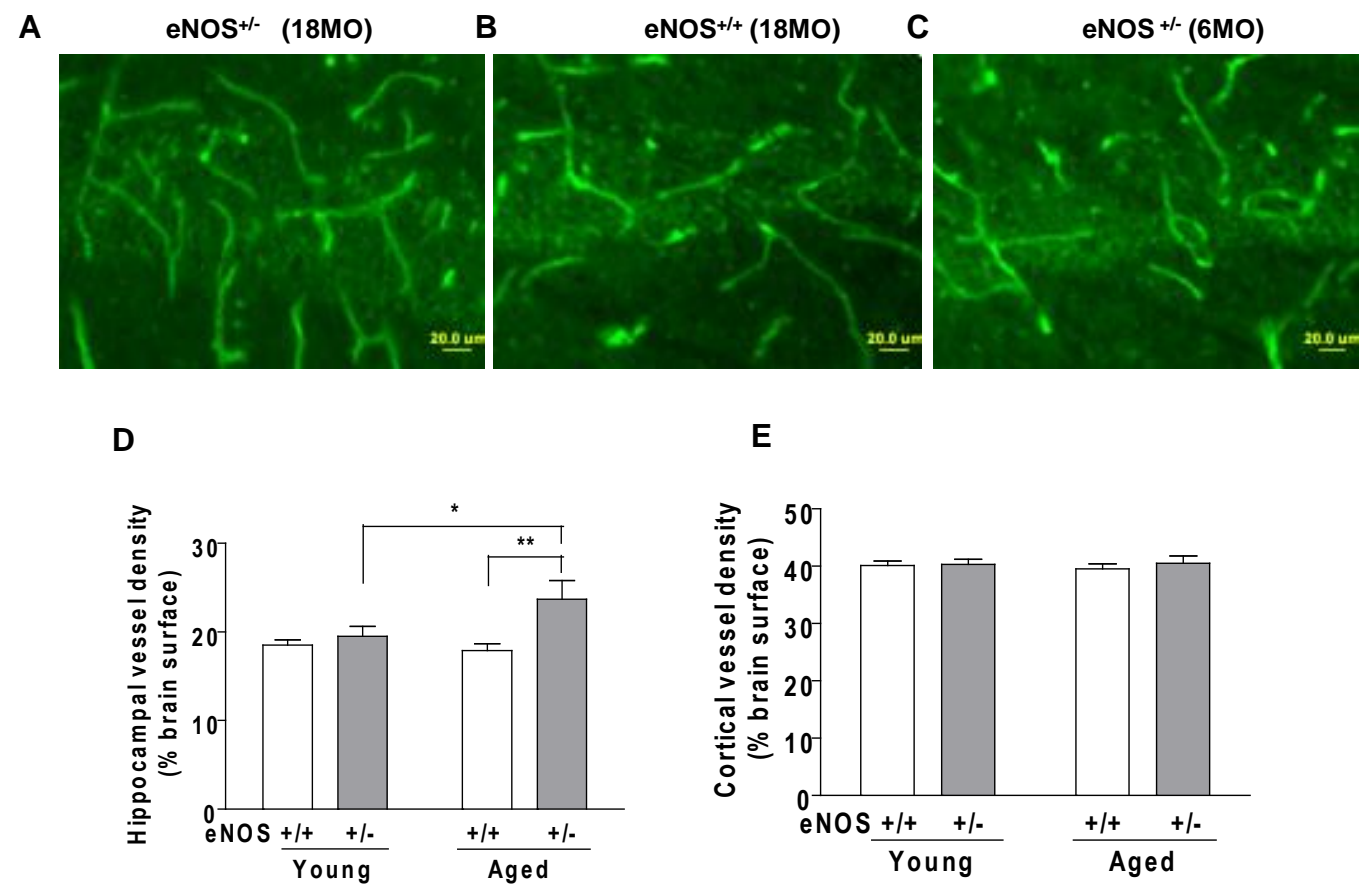

Online Figure 2

Parietal Cortex (mouse IgG)

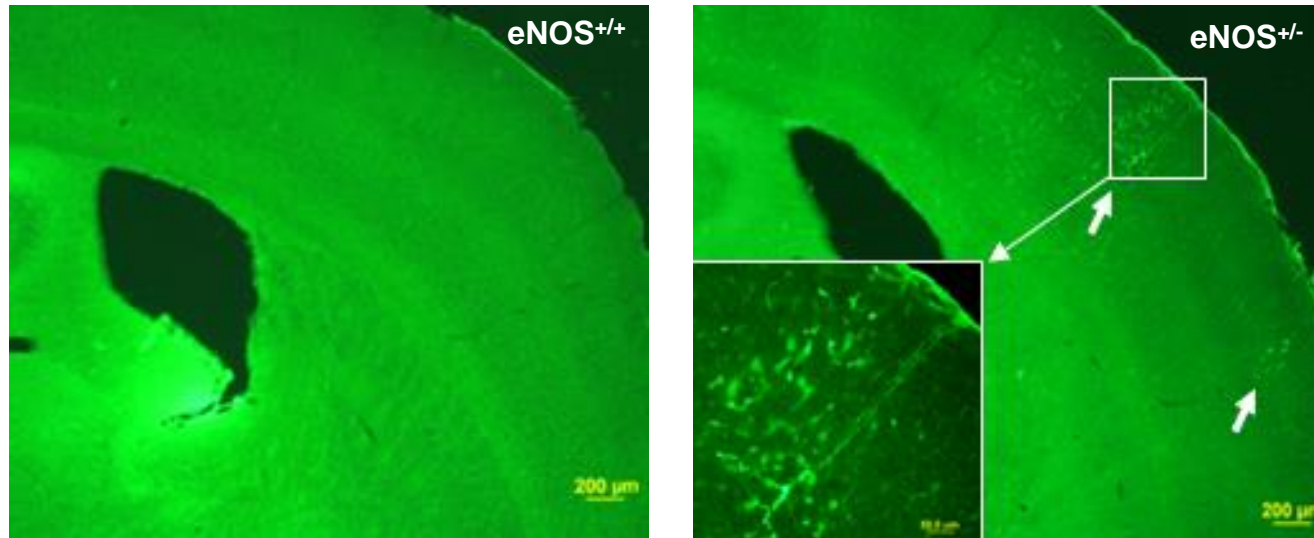

Online Figure 3

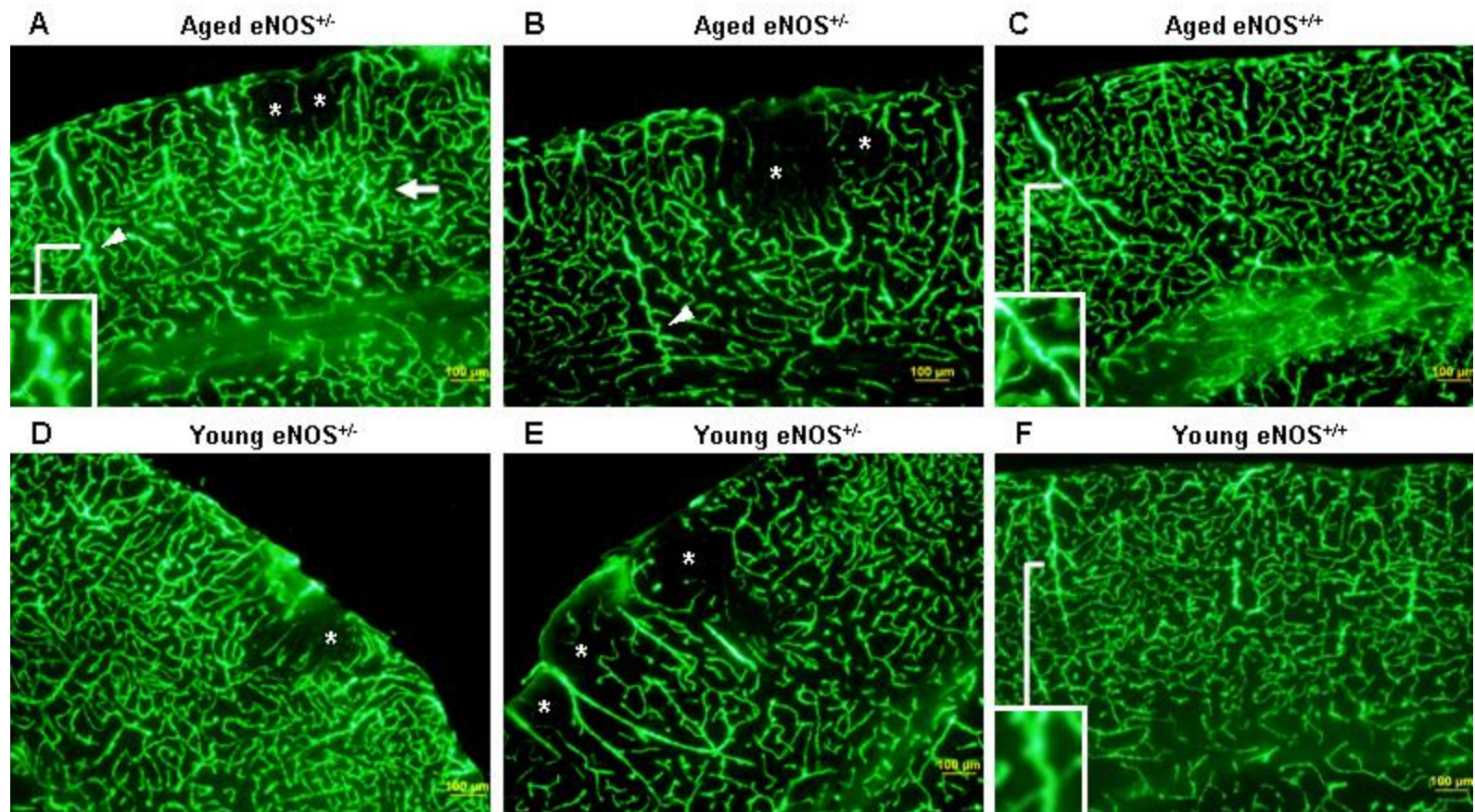

Supplement: Additional file 1: Figure S1. — Cerebral microvessel density in eNOS+/- mice. A-C: Representative images of immunofluorescent staining with anti-CD31 (an endothelial cell marker) antibodies showing vascular hyperplasia in hippocampal CA1 region in aged (18-month-old) eNOS+/- mice (A) compared with either their littermate wild-type (eNOS+/+) mice (B) or young (6-month-old) eNOS+/- mice (C). Scale bar = 20 μm. D and E) Quantification of CD31-positive microvessel density in 4 sections with both hippocampus and cerebral cortex taken at 0.6 mm intervals in young (6-month-old) and aged mice. Results are expressed as mean ± SEM. *P < 0.005; **P < 0.0005. n = 5–6 animals each genotype. Figure S2. Vascular extravasations of serum IgG in 18-month-old eNOS+/- mice. Representative images of mouse IgG immunohistochemistry showing leakage areas framed by white box indicating extravasated mouse serum IgG (green). Scale bars: 200 μm (inset, 50 μm). Figure S3. Cerebral infarction and tortuous cortical arterioles in heterozygous eNOS+/- mice. Cerebral fluorescein isothiocyanate (FITC)-dextran (green) angiographic micrograms showing cerebral microinfarcts/nonperfusion areas (A, B, D, and E. asterisks) in parietal and parietotemporal cortexes with or without surrounding vascular dilation and/or hyperplasia (arrows) in aged (18-month-old) and young (6-month-old) eNOS+/- mice. Note that cortical penetrating arterioles are more tortuous in aged eNOS+/- mice (A and B, arrowheads and inset) compared with their littermate wild-type (eNOS+/+) mice (C and inset) and young eNOS+/- (E) and eNOS+/+ (F and inset) mice. Scale bar = 100 μm. [file 13024_2015_20_MOESM1_ESM.pdf]
